# Baicalein Mediates Mitochondrial Autophagy via miR-30b and the NIX/BNIP3 Signaling Pathway in Parkinson's Disease

**DOI:** 10.1155/2021/2319412

**Published:** 2021-08-18

**Authors:** Min Chen, Li Peng, Ping Gong, Xiaoli Zheng, Tao Sun, Xiaoqiao Zhang, Jiangtao Huo

**Affiliations:** ^1^Department of Geriatrics, Taihe Hospital, Hubei University of Medicine, Shiyan, Hubei, China; ^2^Department of Surgery, Traditional Chinese Medicine Hospital of Qiandongnan Miao and Dong Autonomous Prefecture, Kaili 556000, Guizhou, China

## Abstract

Parkinson's disease (PD) is regarded as a severe neurodegenerative disorder. Baicalein is involved in the treatment of PD. This study explored the mechanism of baicalein in PD. The PD rat model was established using 6-hydroxydopamine. The neurologic score, dopamine (DA) content, apoptotic cells, and neuronal damage were evaluated after rats were treated with baicalein. Autophagy in PD rats was inhibited using 3-methyladenine (3-MA). The mitochondrial membrane potential (MMP) and autophagy-related proteins (LC3, P62) were detected. Next, agomiR-30b was transfected into PD rats. The targeting relation between miR-30b and NIX was predicted and verified. Then, sh-NIX was transfected into PD rats, and the effects of miR-30b and NIX on MMP, LC3, and P62 were assessed. When miR-30b was overexpressed using agomiR-30b, the NIX and BNIP3 levels were detected. Baicalein increased the neurological score and restored DA content, neurons, MMP, and mitochondrial autophagy protein levels. Baicalein inhibited miR-30b expression and miR-30b targeted NIX. miR-30b upregulation or NIX silencing reversed the effect of baicalein on MMP and mitochondrial autophagy. Baicalein upregulated NIX and BNIP3 expressions, while miR-30b overexpression inhibited NIX and BNIP3 expressions. In summary, baicalein mediated mitochondrial autophagy and restored neuronal activity by downregulating miR-30b and activating the NIX/BNIP3 pathway, thus protecting against PD.

## 1. Introduction

Parkinson's disease (PD) is a progressive neurodegenerative disorder that is characterized by motor and nonmotor symptoms [1]. It is one of the most common neurodegenerative disease affecting 1 to 2 out of 1000 of the population worldwide at any time [2]. PD is caused by the degeneration or the pathophysiologic loss of dopaminergic neurons in midbrain substantial nigra and neuronal Lewy body development, which is related to the risk factors such as aging, the family history, environmental chemicals, and pesticide exposure [3]. The complexity of PD brings clinical challenges, including a difficulty in the early stage to make a definitive diagnosis, a difficulty in management at the later stages, and no treatments that can slow the progression of neurodegeneration [4]. Mitochondria are the cell movement energy center that participate in physiological functions, maintenance of metabolism balance, and homeostasis [5]. Mitochondria perform various functions such as producing several biosynthetic intermediates and contributing to the cellular stress responses, including apoptosis and autophagy [6]. Mitochondrial autophagy is a core cellular activity and insufficient autophagy of mitochondrial leads to various aging-related pathologies, especially PD [5, 7, 8]. At present, the most widely used method for autophagy flow detection is to detect the protein level of LC3 by Western blot. In mammalian cells, the total amount of LC3 usually does not have a huge fluctuation, and generally only LC3-II/LC3-I conversion occurs. Therefore, it is generally believed that the increase of LC3-II content represents the activation of autophagy flow, and the decrease of LC3-II content represents the inhibition of autophagy [9]. P62 is a multifunctional protein, which plays an important role in autophagy. It consists of many domains and can bind to different proteins, such as UBA, LIr, and PB1 domains. As p62 is a proteolytic substrate in autophagy, the number of p62 in cells decreases with the increase of autophagy [10]. As a mitochondrial autophagy receptor protein, nip3-like protein *X* (NIX) is involved in the process of autophagy, and although mitochondrial autophagy is mainly mediated by the PINK/Parkin pathway, a previous study has shown that NIX provides a new pathway for autophagy [11]. Further study is needed to explore the pathogenesis of PD in terms of mitochondrial autophagy so as to provide new ideas for clinical treatment.

There are growing interests in developing effective neuroprotective agents, especially from the natural sources [12]. Baicalein is the main active flavonoid of Scutellaria baicalensis, which is reported to possess a variety of pharmacological properties, including reducing oxidative stress, inflammation, apoptosis, and excitotoxicity and stimulating differentiation action and neurogenesis, which invest baicalein with therapeutic potential for PD [13]. It is also reported that baicalein can promote cardiomyocyte mitochondrial autophagy [14].

Baicalein is a natural compound which exerts the anticancer effects by altering microRNA (miRNA) expression [15]. miRNAs have been reported to be a novel type of regulators of cell proliferation, metabolism, and apoptosis [16]. Importantly, miRNAs play a vital role in PD progression, including the diagnosis, pathogenesis, and treatment of PD [17]. miR-30b is identified to be involved in certain kinds of cancers [16]. miR-30b is also involved in neuronal diseases such as oxaliplatin-induced neuropathic pain and is expected to be a novel therapeutic target for the treatment of the neuropathic pain induced by oxaliplatin [18]. Moreover, the effect of miR-30b works on the *α*-synuclein aggregation of PD [19]. miR-30b-5p is significantly overexpressed in the treated patients with PD [20]. miR-30b inhibits the autophagy to protect against the hepatic ischemia-reperfusion injury [21]. But the effect of miR-30b on mitochondrial autophagy in PD is less studied.

However, whether baicalein can regulate miR-30b and mitochondrial autophagy, thus protecting against PD, has not been reported. The aim of this study is to investigate the specific mechanism of baicalein on PD so as to provide new ideas for the treatment of PD.

## 2. Materials and Methods

### 2.1. Ethics Statement

All procedures were authorized by the academic Ethics committee of Taihe Hospital. The experiment was carried out in strict accordance with the guidelines for the management and use of laboratory animals. All the laboratory procedures were used to reduce the pain of the rats.

### 2.2. Laboratory Animals

Specific pathogen-free (SPF) grade adult male Sprague-Dawley (SD) rats (7–10 weeks old, weighing 230–260 g) were provided by the experimental animal center of Guangzhou Sun Yat-sen University (SCXK (Guangdong) 2016–0029, Guangzhou, China). The rats were raised in a 12 h light/dark cycle at a constant temperature of 22–24°C with 50%–60% humidity and freely available food and water.

### 2.3. Treatment and Grouping of SD Rats

AgomiR-30b, agomiR-NC, sh-NIX, and sh-NC (0.8 nM dissolved in 4 *μ*L phosphate buffer saline (PBS)) were purchased from GenScript Biotechnology Co., Ltd. (Nanjing, Jiangsu, China), and were injected into the lateral ventricle of rats 24 h before PD modeling.

The PD rat model was established by injecting neurotoxin 6-hydroxydopamine (6-OHDA) into the rat brain. After anesthesia with 1% pentobarbital (50 mg/kg), the hair on the top of the rat head was cut off, and the rats were fixed on the stereotaxic apparatus. After disinfection with iodine, the scalp, subcutaneous tissue, and periosteum of the rats were cut. The dental drill was used to drill through the stereotactic skull accurately without damaging dura mater. The 6-OHDA solution (8 mg, 4 mL, dissolved in 0.02% ascorbic acid saline, Sigma-Aldrich, St. Louis, MO, USA) was injected into the left medial forebrain bundle (4.3 mm caudal to the bregma, 4.3 mm lateral to the midline, and 8.2 mm below the dural surface) at a rate of 0.5 mL/min. The administration of 6-OHDA was performed using a 10 mL Hamilton microliter syringe, and the syringe needle was retracted slowly after staying in the brain for 5 min to prevent backflow along the injection path. After 3 weeks of modeling, baicalein (100 mg/kg) or 3-methyladenine (3-MA) (100 mg/kg) was intraperitoneally injected into the rats every other day for consecutive 7 times. The tissues used in the following experiments were collected after euthanasia by intraperitoneal injection of 1% pentobarbital (800 mg/kg).

The rats were assigned to the normal group (normal SD rats), sham group (all modeling, exposure, puncture, and other surgical procedures were performed with injection of the same amount of 0.02% ascorbic acid normal saline but without injection of 6-OHDA), PD group (PD model operation was performed), PD + Bai group (baicalein was intraperitoneally injected after PD modeling), PD + Bai + 3-MA group (baicalein and autophagy inhibitor 3-MA were intraperitoneally injected after PD modeling), PD + Bai + agomiR-NC group (agomir-NC was injected into the lateral ventricles of the rats before PD modeling, and baicalein was intraperitoneally injected after PD modeling), PD + Bai + agomiR-30b group (agomiR-30b was injected into the lateral ventricles of the rats before PD modeling, and baicalein was intraperitoneally injected after PD modeling), PD + Bai + sh-NC group (sh-NC was injected into the lateral ventricles of the rats before PD modeling, and baicalein was intraperitoneally injected after PD modeling), and PD + Bai + sh-NIX group (sh-NIX was injected into the lateral ventricles of the rats before PD modeling, and baicalein was intraperitoneally injected after PD modeling). Each group had 12 rats, with 6 for fluorescence staining and 6 for protein detection.

### 2.4. Neurological Score

In this study, all behavioral tests were carried out in the daytime, and adaptive training was carried out three days in advance to avoid anxiety and panic in rats and affecting the experimental results.

In the rotarod test, the rats were placed on a roller rotating at 4 rollings/second and then the roller speed was gradually adjusted with the acceleration of 0.3 rollings/second and the timing started. When the rats fell off the roller, the timing was stopped and the time from the beginning of acceleration to falling was recorded. The process was tested every 1 min for totally 5 times, and the mean value was taken as the final test result.

In the grid test, the rats were placed on a horizontal metal mesh (12 cm × 12 cm, 1 cm apart). The metal mesh device was turned 180 degrees and the timing started after all 4 paws of the rats grasped the metal mesh. The timing was stopped when all four paws fell off the grid. When the hanging time exceeded 180 s, it was recorded as 180 s. The process was tested every 1 min for totally 5 times, and the mean value was taken as the final test result.

### 2.5. Detection of Dopamine (DA) by High Performance Liquid Chromatography-Electrical Chemistry (HPLC-EC)

The brain striatum samples were isolated into an Eppendorf tube and added with 0.1 m/L perchloric acid solution to completely break the tissue cells. The samples were placed into a precooled centrifuge and centrifuged at 4°C and 14000 g for 20 min. An appropriate amount of tissue supernatant was diluted (mobile phase: including 3 mM 1-octanesulfonic acid, 100 mM sodium nitrate, 85 mM citric acid, 0.2 mM ethylenediaminetetraacetic acid, and 8% methanol) and detected on the machine, and the content of DA was calculated.

### 2.6. TUNEL Staining and Fluoro-Jade B (FJB) Staining

The brain tissue was fixed with 4% paraformaldehyde for 24 h and successively dehydrated in 15% and 30% sucrose. After embedding with optimum cutting temperature (OCT) freezing medium, the tissue was cut into frozen sections at 15 mm. After staining according to the instructions of the TUNEL kit (Abcam Inc., Cambridge, MA, USA), the sections were incubated with 4′,6-diamidino-2-phenylindole (DAPI) for 5 min and observed under Leica fluorescence microscope (×200, Leica, Wetzlar, Germany). The injured neurons were observed by FJB (Histo-chem Inc., Jefferson, AR, USA) staining. The sections were incubated firstly in 0.06% KMnO_4_ and then in 0.001% FJB. The Hamamatsu Nanozoomer 2.0 HT (Olympus Corporation, Tokyo, Japan) was used to obtain the images.

### 2.7. Detection of Mitochondrial Membrane Potential (MMP)

The MMP was determined by JC-1 staining (Thermo Fisher Scientific, San Jose, CA, USA) according to the manufacturer's instructions. The tissue cells were incubated with JC-1 solution in the dark at 37°C for 30 min and the supernatant was removed. The cells were washed twice with PBS and imaged under fluorescence microscope (×100, Leica).

### 2.8. Western Blot (WB)

The tissue was homogenized at 1 : 10 (w/v) in homogenate buffer (including 20 mM Tris-HCL, 150 mM Nacl, 1 mM EDTA, 2.5 mM sodium pyrophosphate, 1 mM b-glycerophosphate, 1 mM sodium vanadate, 1% Triton X-100 and 1 mg/mL leupeptin hemisulfate salt; pH 7.5) and added with protease inhibitor cocktail. After the homogenate was centrifuged at 12000 g for 10 min, the protein lysate was quantified using the bicinchoninic acid (Bio-Rad, Hercules, CA, USA) method. Protein was isolated by 10% sodium dodecyl sulfate polyacrylamide gel electrophoresis (SDS-PAGE) (Beyotime, Shanghai, China) and transferred to polyvinylidene fluoride (PVDF) membranes (Millipore, Billerica, MA, USA). After sealing in PBS buffer containing 5% skim milk powder at room temperature for 2 h, the membranes were incubated with primary antibodies at 4°C overnight, and then the membranes were incubated with the secondary antibody IgG (1 : 5000, ab205718, Abcam, UK) at room temperature for 2 h. The enhanced chemiluminescence kit (Thermo Fisher Scientific) was used to detect the protein bands and Image J software was used for quantitative analysis. The primary antibodies included LC3 (1 : 2000, ab192890, Abcam), P62 (1 : 1000, ab109012, Abcam), NIX (1 : 1000, ab109414, Abcam), BNIP3 (1 : 1000, ab109362, Abcam), and *β*-actin (1 : 1000, ab8227, Abcam).

### 2.9. Reverse Transcription Quantitative Polymerase Chain Reaction (RT-qPCR)

TRIzol (Invitrogen, Carlsbad, CA, USA) was used to extract total RNA from tissue and homogenate. The cDNA template was synthesized by extracted RNA using TaqMan microRNA Reverse Transcription kit (Applied Biosystems, Foster City, CA, USA). Quantitative PCR amplification was performed according to SYBR Premix Ex Taq. GAPDH or U6 was used to be internal parameter and the 2^−△△Ct^ method was used to calculate the relative expressions of miR-30b and NIX. Each sample was independently tested 3 times. Primer sequences are shown in Table 1.

### 2.10. Dual-Luciferase Assay

The binding sites of miR-30b and NIX were analyzed by the bioinformatics online software TargetScan (http://www.targetscan.org/vert_72/). The wild-type (NIX-wt) and mutant-type (NIX-mut) luciferase plasmids were constructed by cloning the binding and mutated sequences into the luciferase vector pGL3 (Promega, Madison, WI, USA). The 293 T cells (ATCC, Manassas, VA, USA) were seeded into 6-well plates (2 × 10^5^ cells/well) and incubated for 24 h. The constructed luciferase vectors were cotransfected with mimic NC or miR-30b mimic (Shanghai Genechem Co., Ltd., Shanghai, China) (miRNA-mimic 20 nM) into 293T cells using LiPofectamine 2000 (11668–019, Invitrogen, Carlsbad, CA, USA) according to the provided instructions. The luciferase activity was evaluated by Dual-Lucy Assay kit (Solarbio, Beijing, China) after 24 h. The cell experiment was repeated 3 times independently.

### 2.11. Statistical Analysis

SPSS21.0 statistical software (IBM Corp. Armonk, NY, USA) was used for statistical analysis of the data. Kolmogorov–SmiRnov test showed that the data were in normal distribution and expressed as mean ± standard deviation. Independent *t*-test was used for comparisons between two groups and one-way analysis of variance (ANOVA) was used for comparisons among multigroups. Tukey's multiple comparisons test was used for the post hoc test. *P* value was obtained by a bilateral test, where *P* < 0.05 was indicative of statistical significance.

## 3. Results

### 3.1. Baicalein Played a Protective Role in Neuronal Damage of PD Rats

**Figure 1:**
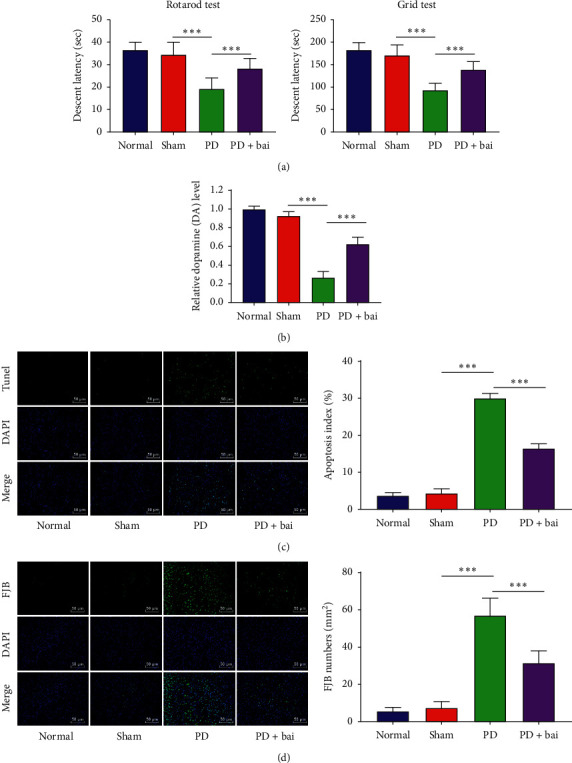
Baicalein played a protective role in PD rat model. PD model operation was performed in SD rats, and the protective effect of baicalein on PD rats was observed after baicalein treatment. (a) Neurological score; (b) DA content was detected by HPLC-EC; (c) apoptosis was detected by TUNEL staining; (d) neuronal degeneration was detected by Fluoro Jade B staining. *N* = 6. The data in the figure are all measurement data and the data are expressed as mean ± standard deviation; one-way ANOVA was used for variance analysis; Tukey's multiple comparisons test was used for post hoc test. ^*∗∗∗*^*P* < 0.001.

Baicalein is a natural bioactive flavone extracted from the root of Scutellaria baicalensis, which is mainly used as a natural neuroprotective agent as phytochemicals show high efficiency and low side effects in various *in vitro* and *in vivo* studies [12]. In order to determine the effect of baicalein on the PD rat model, the neurological scores of rats in the sham group, PD group, and PD + Bai group were evaluated (Figure 1(a)). It was found that the rats in the PD group showed severe neurological deficits, and the related motor and sensory coordination was decreased (*P* < 0.001), while the intervention of baicalein alleviated neurobehavioral defects caused by surgery-induced PD to some extent. The content of DA was detected by HPLC-EC (Figure 1(b)). It was found that the DA content in striatum of rats in the PD group was significantly decreased (*P* < 0.001), while baicalein restored the content of DA to some extent. The apoptosis was detected by TUNEL staining (Figure 1(c)). Compared with the sham group, the apoptosis of rats in the PD group was increased significantly (*P* < 0.001), while baicalein reduced the apoptosis. The injured neurons were detected by FJB staining (Figure 1(d)). Compared with the sham group, the degeneration of neurons of rats in the PD group was increased significantly (*P* < 0.001), while baicalein alleviated the damage of neurons. These results suggested that baicalein had a protective effect on the PD rat model.

### 3.2. Baicalein Played a Protective Role in PD Rat Neuronal Damage through Promoting to Chondrial Autophagy

**Figure 2:**
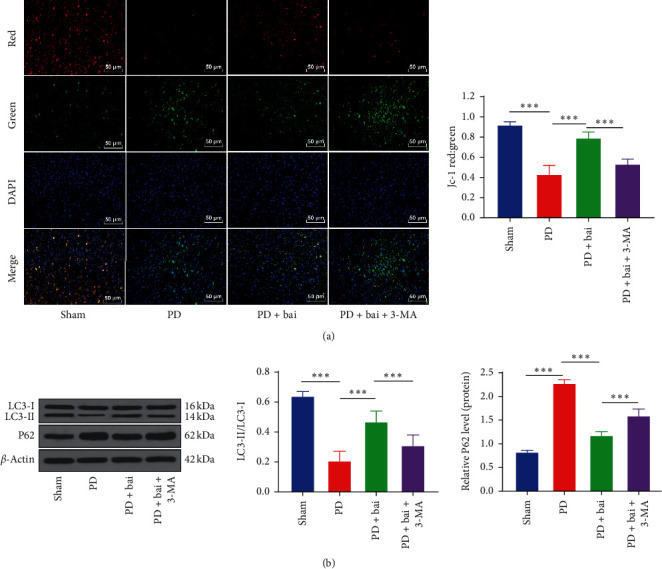
Baicalein played a protective role through promoting mitochondrial autophagy. PD rats were joint intervened by baicalein and autophagy inhibitor 3-MA and the effect of baicalein on mitochondrial autophagy was observed. (a) Mitochondrial membrane potential was detected by JC-1 fluorescence staining. (b) The expressions of LC3 and P62 were detected by WB. *N* = 6. The data in the figure are all measurement data and the data are expressed as mean ± standard deviation; one-way ANOVA was used for variance analysis; Tukey's multiple comparisons test was used for post hoc test. ^*∗∗∗*^*P* < 0.001.

Mitochondria are vital in cell bioenergy and apoptosis. Its structure and function are closely related to neurodegenerative diseases [8]. Mitochondrial autophagy plays an important role in the disorder of mitochondrial structure and function caused by neurodegenerative diseases [5]. Therefore, we speculated that the protection of baicalein on PD rats was closely related to mitochondrial autophagy. The MMP in the sham group, PD group, PD + Bai group, and PD + 3-MA + Bai group was measured (Figure 2(a)). It was found that PD decreased MMP (*P* < 0.001). As the MMP was decreased, it was easy to detect the fluorescence changing from red to green. After the intervention of baicalein, the situation was improved and the MMP returned to a certain level; compared with baicalein intervention, the autophagy inhibitor 3-MA treatment reversed the benign effect of baicalein (*P* < 0.001). In order to further verify the results above, the expressions of LC3 and P62 in the sham group, PD group, PD + Bai group, and PD + Bai + 3-MA group were compared (Figure 2(b)). Compared with the sham group, the LC3-II/LC3-I in PD group was downregulated, while the expression of P62 was upregulated (*P* < 0.001). Compared with the PD group, the LC3-II/LC3-I in the baicalein group was upregulated and the expression of P62 was downregulated (*P* < 0.001). Compared with the baicalein group, 3-MA treatment annulled the effect of baicalein (*P* < 0.001). The levels of autophagy-related proteins LC3 and P62 corresponded to the results of MMP detection, suggesting that baicalein played a protective role by promoting mitochondrial autophagy.

### 3.3. Baicalein Promoted Mitochondrial Autophagy by Downregulating miR-30b Expression in PD Rats

**Figure 3:**
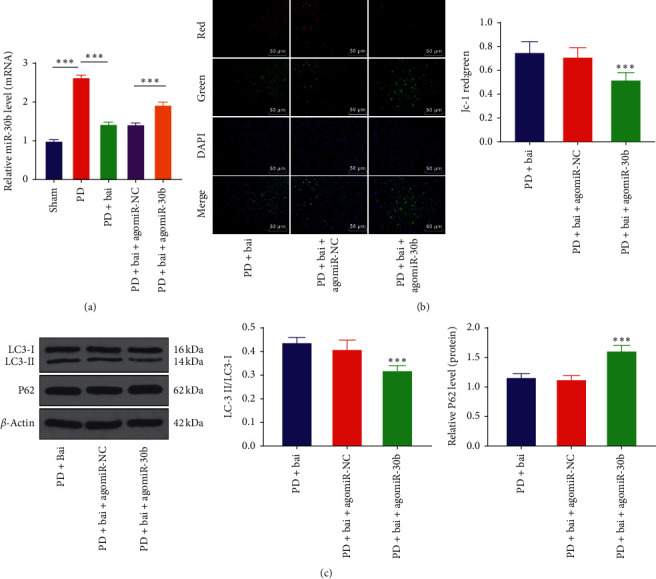
The upregulation of miR-30b reversed the promotive effect of baicalein on mitochondrial autophagy. The agomiR-30b was transfected into PD rats and the effect of overexpression of miR-30b on baicalein induced mitochondrial autophagy was observed. (a) The expression of miR-30b was detected by RT-qPCR. (b) Mitochondrial membrane potential was detected by JC-1 fluorescence staining. (c) The expressions of LC3 and P62 were detected by WB. *N* = 6. The data in the figure are all measurement data and the data are expressed as mean ± standard deviation; one-way ANOVA was used for variance analysis; Tukey's multiple comparisons test was used for post hoc test. ^*∗∗∗*^*P* < 0.001.

It is reported that miR-30b can inhibit autophagy [22]. Baicalein protected PD rats by mitochondrial autophagy. Therefore, we speculated that baicalein could affect the expression of miR-30b. To verify this hypothesis, the expression of miR-30b was detected by RT-qPCR (Figure 3(a)). It was found that the expression of miR-30b was upregulated significantly in the PD group (*P* < 0.001), while baicalein inhibited the expression of miR-30b in PD rats (*P* < 0.001). Subsequently, the miR-30b was overexpressed in PD rats using agomiR-30b to observe its effect on baicalein-induced mitochondrial autophagy (Figure 3(a)). It was found that the overexpression of miR-30b partially reversed the effect of baicalein on mitochondrial autophagy in PD rats (Figures 3(b) and 3(c)). The results suggested that baicalein promoted mitochondrial autophagy by downregulating miR-30b.

### 3.4. miR-30b Targeted NIX

**Figure 4:**
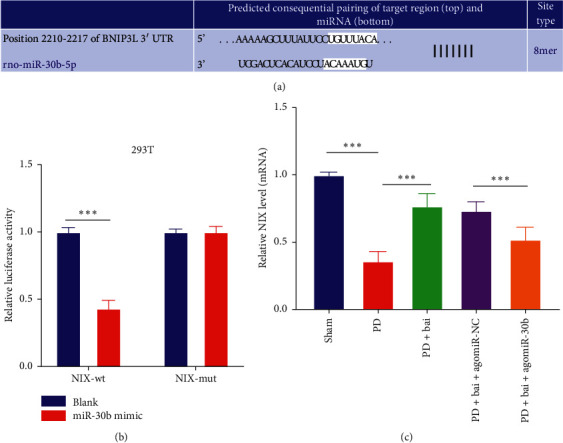
miR-30b targeted NIX. (a) The binding sites between miR-30b and NIX were predicted by TargetScan website. (b) The target binding relationship between miR-30b and NIX was verified by dual-luciferase assay. (c) The expression of NIX was detected by RT-qPCR. Three independent repeated cell tests were performed. *N* = 6. The data in the figure are all measurement data and the data are expressed as mean ± standard deviation; two-way ANOVA (Figure (b)) and one-way ANOVA (Figure (c)) were used for variance analysis; Tukey's multiple comparisons test was used for post hoc test. ^*∗∗∗*^*P* < 0.001.

To further understand the potential mechanism of baicalein in regulating miR-30b, the downstream target genes of miR-30b were predicted by the TargetScan website (http://www.targetscan.org/vert_72/). Then, NIX was obtained (Figure 4(a)). NIX was reported to play a protective role by promoting mitochondrial autophagy in neurodegenerative diseases [23]. The target binding relationship between miR-30b and NIX was verified by dual-luciferase assay in 293T cells (Figure 4(b)). Finally, the relative expression of NIX in rats was detected by RT-qPCR (Figure 4(c)). It was found that when the level of miR-30b was upregulated, the mRNA level of NIX was downregulated, which suggested that miR-30b could target NIX.

### 3.5. Downregulating NIX Reversed Baicalein-Induced Mitochondrial Autophagy

**Figure 5:**
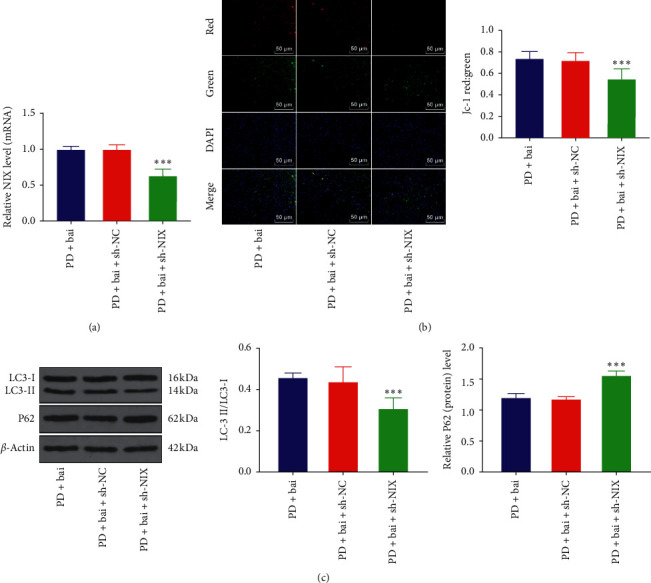
Downregulation of NIX reversed baicalein induced mitochondrial autophagy. The effect of NIX on baicalein induced mitochondrial autophagy was observed through inhibiting the expression of NIX in PD rats by sh-NIX. (a) The expression of NIX was detected by RT-qPCR. (b) Mitochondrial membrane potential was detected by JC-1 fluorescence staining. (c) The expressions of LC3 and P62 were detected by WB. *N* = 6. The data in the figure are all measurement data and the data are expressed as mean ± standard deviation; one-way ANOVA was used for variance analysis; Tukey's multiple comparisons test was used for post hoc test. ^*∗∗∗*^*P* < 0.001.

To study the mechanism of NIX on mitochondrial autophagy, the expression of NIX was downregulated in PD rats by intracerebroventricular injection of sh-NIX (Figure 5(a)). Subsequently, the MMP was measured in each group (Figure 5(b)) and LC3, P62 was detected by WB (Figure 5(c)). The inhibition of NIX partially reversed the effect of baicalein on upregulating MMP and reversed the effects of baicalein on upregulating LC3-II/LC3-I and downregulating P62 expressions (*P* < 0.001), suggesting that the downregulation of NIX reversed the effect of baicalein on promoting the mitochondrial autophagy.

### 3.6. Baicalein Mediated Mitochondrial Autophagy via miR-30b and the NIX/BNIP3 Signaling Pathway

**Figure 6:**
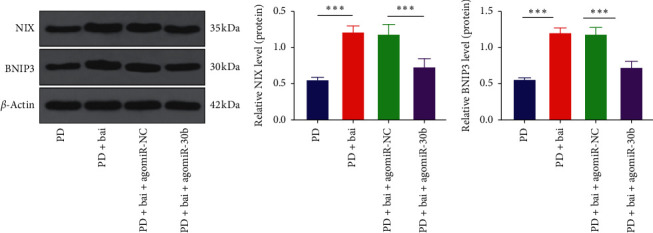
Baicalein mediated mitochondrial autophagy via miR-30b and the NIX/BNIP3 signaling pathway. The effect of miR-30b on the NIX/BNIP3 signaling pathway was observed by transfecting agomir-30b in PD rats. (a) The expressions of NIX and BNIP3 were detected by WB. *N* = 6. The data in the figure are all measurement data and the data are expressed as mean ± standard deviation; one-way ANOVA was used for variance analysis; Tukey's multiple comparisons test was used for post hoc test. ^*∗∗∗*^*P* < 0.001.

The NIX/BNIP3 signaling pathway is reported to be an important pathway to mediate mitochondrial autophagy [24]. It was confirmed in our previous experiments that baicalein played a role in mitochondrial autophagy by regulating NIX via miR-30b. Therefore, we speculated that baicalein regulated mitochondrial autophagy via the NIX/BNIP3 signaling pathway. To verify this hypothesis, the levels of NIX and BNIP3 in the PD group, PD + Bai group, PD + Bai + agomiR-NC group, and PD + Bai + agomiR-30b group were detected by WB (Figure 6(a)). It was found that baicalein could upregulate the levels of NIX and BNIP3 in PD rats (*P* < 0.001), and overexpression of miR-30b reversed the effect of baicalein on upregulating the levels of NIX and BNIP3 (*P* < 0.001), suggesting that baicalein had an effect on mitochondrial autophagy by miR-30b and the NIX/BNIP3 signaling pathway.

## 4. Discussion

PD is the most serious and common neurodegenerative disorder all over the world and the prevalence of it is predicted to increase significantly as population ages [25]. A previous study has demonstrated the neuroprotective effect of baicalein on PD [26]. This study concentrated on the mechanism of baicalein on PD and found that baicalein exerted neuroprotective effects on PD rats by inhibiting miR-30b and promoting mitochondrial autophagy mediated by the NIX/BNIP3 signaling pathway.

In PD, rehabilitation of neurological function is effective in protecting the condition of patient against further worsening [27]. Our results showed that the baicalein treatment alleviated neurobehavioral defects and the damage of neurons in PD rats to some extent. The dopaminergic neurons loss is responsible for the motor symptoms of PD [28]. PD is one of the neurodegenerative disorders related to the downregulation of DA content [29]. This study demonstrated that the baicalein intervention restored the content of DA in PD rats to some extent. High dose of baicalein attenuated the reductions of DA content in striatum significantly [30]. Baicalein is reported to inhibit apoptosis in cardiomyocytes [14]. Our results showed that the apoptosis of PD rats was significantly increased, while baicalein treatment reduced the apoptosis. Consistently, baicalein is reported to possess the pharmacological property of neurogenesis and effect of antiapoptosis [13]. In brief, baicalein played a protective role in PD rats.

Mitochondrial function is an essential indicator of cell health, which is assessed by monitoring the changes in MMP [31]. Mitochondrial autophagy is reported to play an essential role in a variety of neurodegenerative diseases and apoptosis [8]. We speculated that the protective effect of baicalein on PD rats is closely related to mitochondrial autophagy. Our results showed that PD decreased MMP in PD rats. The MMP returned to a certain level after baicalein intervention, while autophagy inhibitor 3-MA reversed the effect of baicalein in PD. LC3 and P62 proteins are used to monitor the autophagic flux [32]. The LC3-II/LC3-I is the indicative of autophagy status [33]. Our results revealed that the LC3-II/LC3-I was downregulated and the P62 expression was upregulated in PD. The LC3-II/LC3-I ratio was upregulated and the P62 expression was downregulated after baicalein intervention. The 3-MA annulled the effect of baicalein in PD. The autophagy-related proteins LC3 and P62 levels were corresponded to the MMP detection results, indicating that baicalein had a protective effect by promoting mitochondrial autophagy. It is consistent with that the LC3-II/LC3-I is increased significantly [34] and P62 expression is decreased in PD [10]. Baicalein decreases the LC3-II/LC3-I in ischemic stroke [35] and increased P62 expression in acute liver injury [36]. Baicalein has been reported to prevent neurotoxicity via restoring the autophagy [37]. In summary, baicalein played a protective role in PD rats through mitochondrial autophagy.

miR-30b is reported to show the effect of inhibiting of autophagy [22]. It was found by our previous experiments that baicalein played a protective role through mitochondrial autophagy. Therefore, we speculated that baicalein could regulate the expression of miR-30b. Our results showed that the miR-30b expression was upregulated in PD rats, while baicalein inhibited the miR-30b expression. The overexpression of miR-30b partially reversed the effect of baicalein on mitochondrial autophagy in PD rats. It is consistent with that a significant upregulation of miR-30b was observed in PD patients [20]. However, there is a little study on the effect of baicalein on the regulation of miR-30. This study initially supported that the baicalein promoted mitochondrial autophagy by downregulating miR-30b.

To further understand the potential mechanism of baicalein on regulating miR-30b, we predicted the downstream target genes of miR-30b. Then, NIX was obtained. NIX restores mitochondrial function and mitochondrial autophagy to protect against PD [38]. The target binding relationship between NIX and miR-30b was verified by dual-luciferase assay. Besides, our results demonstrated that the NIX level was downregulated when the miR-30b level was upregulated. Our study suggested that miR-30b could target NIX. To study the mechanism of NIX on mitochondrial autophagy, we downregulated the NIX expression in PD rats by intracerebroventricular injection of sh-NIX. NIX inhibition partially reversed the effect of baicalein on the upregulation of MMP, the upregulation of LC3-II/LC3-I, and the downregulation of P62 expression. NIX restores the autophagy and function of mitochondrial to protect against PD [11]. Briefly, downregulation of NIX could reverse the effect of baicalein on the mitochondrial autophagy.

The NIX/BNIP3 pathway is identified to be involved in mediating mitochondrial autophagy [24, 39]. Therefore, we speculated that baicalein regulated mitochondrial autophagy via the NIX/BNIP3 signaling pathway. Our results revealed that baicalein upregulated NIX and BNIP3 levels in PD rats and overexpression of miR-30b inhibited the NIX/BNIP3 pathway. NIX/BNIP3L promotes mitochondrial autophagy in PD pathogenesis [40]. Briefly, baicalein exerted the effect on mitochondrial autophagy via miR-30b and the NIX/BNIP3 signaling pathway.

A large number of mitochondria are distributed in synapses and growth cones of neurons, which are of great significance for maintaining the activity of neurons. Due to the risk of mitochondrial damage or aging, timely elimination of abnormal mitochondria through mitochondrial autophagy is necessary to maintain mitochondrial peace and cell homeostasis [41]. Therefore, mitochondrial autophagy may play an important role in the maintenance of nervous system function [42]. Our study showed that baicalein could regulate the expression of miR-30b and mitochondrial autophagy in PD rats, indicating that baicalein might reduce the accumulation of dysfunctional mitochondria by promoting mitochondrial autophagy and make the activity of neurons tend to be stable again. In summary, this study supported that baicalein exerted neuroprotective effect on PD by promoting mitochondrial autophagy through downregulating miR-30b and upregulating the NIX/BNIP3 signaling pathway. However, whether baicalein had regulatory effect on other mitochondrial autophagy signal pathways and whether the regulatory effect of baicalein could provide new ideas for clinical treatment of PD were not deeply studied yet. Further work is needed to study the clinical treatment of PD from the regulation mechanism of baicalein.

## Figures and Tables

**Table 1:** Primer sequence.

Name of primer	Sequences
miR-30b-F	CACCAGCCATGTAAACATCC
miR-30b-R	ATGCTTGTTCTCGTCTCTGT
NIX-F	CAATGTCGTCCCACCTAGTCG
NIX-R	TAGCTCCACCCAGGAACTGTTG
GAPDH-F	CAAGCAACTGTCCCTGAG
GAPDH-R	TAGACAGAAGGTGGCACA
U6–F	ATTGGAACGATACAGAGAAG
U6-R	GGAACGCTTCACGAATTTG

## Data Availability

All the data generated or analyzed during this study are included in this published article.
